# Underestimating the Alcohol Content of a Glass of Wine: The Implications for Estimates of Mortality Risk

**DOI:** 10.1093/alcalc/agw027

**Published:** 2016-09-26

**Authors:** Annie Britton, Darragh O’Neill, Steven Bell

**Affiliations:** Research Department of Epidemiology and Public Health, University College London, 1-19 Torrington Place, London WC1E6BT, UK

## Abstract

**Aims:**

Increases in glass sizes and wine strength over the last 25 years in the UK are likely to have led to an underestimation of alcohol intake in population studies. We explore whether this probable misclassification affects the association between average alcohol intake and risk of mortality from all causes, cardiovascular disease and cancer.

**Methods:**

Self-reported alcohol consumption in 1997–1999 among 7010 men and women in the Whitehall II cohort of British civil servants was linked to the risk of mortality until mid-2015. A conversion factor of 8 g of alcohol per wine glass (1 unit) was compared with a conversion of 16 g per wine glass (2 units).

**Results:**

When applying a higher alcohol content conversion for wine consumption, the proportion of heavy/very heavy drinkers increased from 28% to 41% for men and 15% to 28% for women. There was a significantly increased risk of very heavy drinking compared with moderate drinking for deaths from all causes and cancer before and after change in wine conversion; however, the hazard ratios were reduced when a higher wine conversion was used.

**Conclusions:**

In this population-based study, assuming higher alcohol content in wine glasses changed the estimates of mortality risk. We propose that investigator-led cohorts need to revisit conversion factors based on more accurate estimates of alcohol content in wine glasses. Prospectively, researchers need to collect more detailed information on alcohol including serving sizes and strength.

**Short summary:**

The alcohol content in a wine glass is likely to be underestimated in population surveys as wine strength and serving size have increased in recent years. We demonstrate that in a large cohort study, this underestimation affects estimates of mortality risk. Investigator-led cohorts need to revisit conversion factors based on more accurate estimates of alcohol content in wine glasses.

## Introduction

The chronic harm to health from alcohol consumption in the population is typically determined using findings from longitudinal observational studies with self-reported consumption as the exposure. There are concerns that self-reported consumption underestimates the true amount of alcohol an individual consumes, in part due to underestimating the serving size and/or alcoholic strength of drinks (Boniface and Schelton, 2013; Bellis *et al.*, 2015). Misclassification of alcohol intake has implications firstly for calculating the proportion of the population who drink above low-risk drinking guidelines and secondly for estimating the health risks associated with different levels of consumption (Stead *et al.*, 2013).

In the UK over the past 25 years, there has been an increase in glass size serving and the strength of alcoholic beverages, particularly for wine (Stead *et al.*, 2013), which is likely to have resulted in people underestimating the amount of alcohol that they consume. Similar trends have previously been observed in North America (Kerr *et al.*, 2006). It is estimated that between 1990 and 2007, the average strength of wine increased by 13%, from 11.2% to 12.6% Alcohol by Volume (ABV) (Stead *et al.*, 2013). Since 1995, the legally permitted measure sizes for wine by the glass in the UK have been 125 ml, 175 ml, 250 ml and multiples thereof. Prior to this, no measure size was stipulated, but the 125-ml measure tended to be the most used in licensed premises (Keeling, 1997). Licensees were encouraged to call 175 ml a ‘standard’ glass and 125 ml ‘small’ (Stead *et al.*, 2014), therefore effectively increasing the typical size offered by 50 ml.

In the 1980s and 1990s, the UK Department of Health’s sensible drinking advice defined one glass of wine to be equivalent to 8 g of alcohol (1 unit of alcohol), assuming a 125 ml glass of 8% ABV wine (Department of Health, 1995). This conversion factor of one wine glass being 8 g of alcohol (1 unit) then formed the basis for estimating the alcohol intake in populations and subsequent health risks. A review in 2005 by the Office for National Statistics (ONS) resulted in a revised method by which survey data on drinks consumed was converted into units of alcohol (Goddard, 2007). Since 2006, one glass of wine (size not specified) is assumed to contain 2 units, a small glass (125 ml) is 1.5 units, a medium glass (175 ml) is 2 units and a large glass (250 ml) is 3 units. The impact of the changes in 2006 increased estimates of average weekly consumption in England by 22% for men and 46% for women (Stead *et al.*, 2013).

Investigator-led epidemiological cohort studies in progress prior to this may not have considered updating their conversion factors, and this might have implications for findings linking alcohol to health outcomes (Stead *et al.*, 2013). This has not been explicitly tested before as most previous work focuses on the characteristics of those who under-report but not whether the updated conversion factors also modify risk associations (Boniface *et al.*, 2014).

In this paper, we extend work in this area further by exploring whether the likely misclassification in alcohol content in wine affects the association between average alcohol intake and risk of death from all causes, cardiovascular disease (CVD) and cancer.

## Methods

### Study population

Data were drawn from the Whitehall II cohort study of British civil servants (Marmot and Brunner, 2005). The study started in 1985 and consisted of 10,308 (6895 men) participants aged 35–55 years. Data used in this investigation come from the Phase 5 data collection (1997–1999), representing the first measurement occasion after the legal introduction of standardized wine serving sizes in the UK and the shift from 125 to 175 ml glasses becoming the normative standard serving size. Participants were aged 47 to 67 years, and the final sample size was 7010 (4953 men) after excluding individuals who did not take part at Phase 5 as well as those with missing values of alcohol consumption and covariates.

The University College London Medical School Committee on the ethics of human research approved the Whitehall II study. Whitehall II data, protocols and other metadata are available to *bona fide* researchers for research purposes (data sharing policy is available at http://www.ucl.ac.uk/whitehallII/data-sharing).

### Assessment of alcohol consumption

Participants were asked to report the number of alcoholic drinks they had consumed in the previous week, providing information separately for beer/cider (in pints), wine (in glasses) and spirits (in measures). Initially drinks were converted into grams of ethanol assuming 16 g for each pint of beer/cider and 8 g for each measure of spirits or glass of wine. The sum of these converted measurements was then used to define total weekly volume of alcohol intake in grams. We refer to this approach as the ‘pre-change’ conversion. In order to reflect that 8 g of ethanol is likely to be an underestimate of the alcohol content in a glass of wine, we then doubled this to 16 g of ethanol. This represents 2 UK units of alcohol and is in line with the ONS 2005 review (Goddard, 2007). Our decision was driven not only by the change in standard serving size to 175 ml glasses but also by the changes in the average ABV content of wine during this period as calculated by Stead *et al.* (2013). The modal ABV of wine being 11.5% during 1997–1999, and 11.5% ABV × 175 ml = 2.01 UK units per glass. We then re-estimated the total weekly volume of alcohol and called this the ‘post-change’ conversion. We were not able to adjust beer as information was only collected on pints (not strength or bottle size).

We constructed categories of alcohol consumption based on UK guidelines of sensible drinking at that time (Department of Health, 1995). These were none, moderate (within guidelines of 1–168 g (1–21 units) of ethanol per week for men and 1–112 g (1–14 units) for women), heavy (169–407 g (22–50 units) for men and 113–287 (15–35 units) for women) and very heavy (408+g (51+ units) for men and 288+g (36+ units) for women) plus a former drinker category (current non-drinker but indicated alcohol consumption in the past).

### Assessment of mortality

Participants were traced for mortality through the national mortality register kept by the National Health Services (NHS) Central Registry using the NHS identification number assigned to each British citizen. Deaths from all causes, CVD and cancer were traced until June 2015.

### Covariates

Covariates included demographic characteristics, namely age, sex, ethnicity (white or non-white) and socio-economic position (SEP; defined using either current or last recorded civil service employment grade as high, intermediate or low), as well as behavioural/lifestyle factors such as smoking status (never, ex- and current), physical activity (lowest sex-specific quartile of combined hours of moderate and vigorous physical activity defined as ‘physically inactive’) and diet quality (classified as poor or good using three questions on the type of milk and bread participants usually consumed and their frequency of fruit and vegetable intake). Detailed descriptions of these covariates have been previously published (Britton and Marmot, 2004).

### Statistical analysis

Cox proportional hazard models were used to estimate hazard ratios (HRs) and associated 95% confidence intervals (95% CI) of association between drinking category and mortality from all causes, CVD and cancer with moderate drinkers as the reference group. We verified the proportional hazards assumption using Schoenfeld residuals. We calculated the percentage attenuation/amplification of the effect observed and examined heterogeneity in estimates pre- and post-conversion using the *I* ^2^ statistic. Analyses were conducted using Stata 14.1.

## Results

### Descriptive statistics

**Fig. 1.**
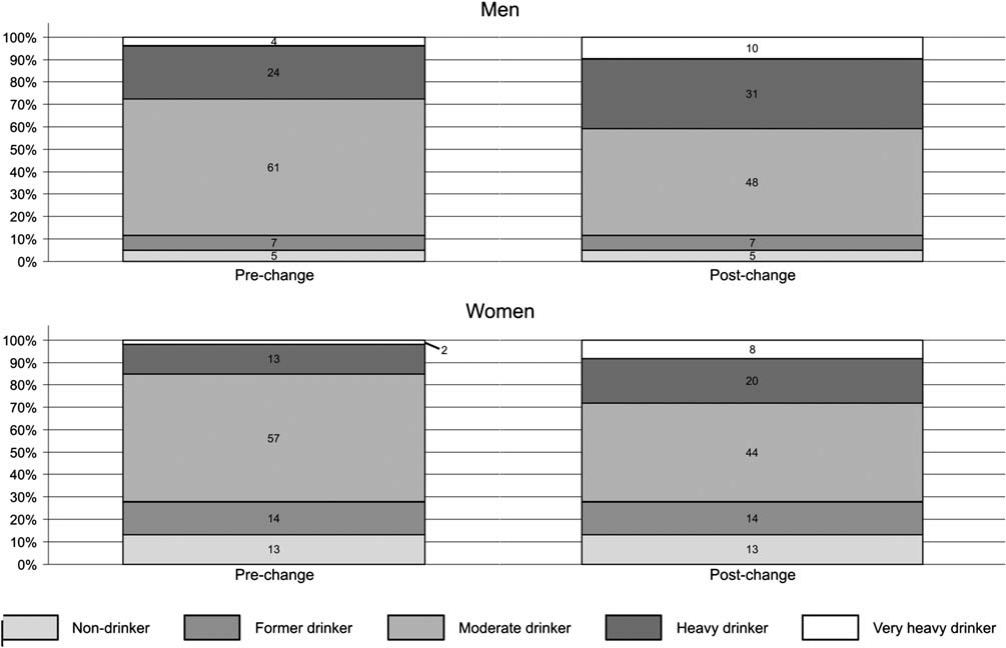
Change in the proportion of men and women in each drinking category pre- and post-change in wine conversion.

When a glass of wine was considered to contain 16 g of alcohol as opposed to 8 g of alcohol, there was a substantial shift in the proportion of drinkers who were classified as moderate into the heavier categories (Fig. 1). For men, the proportion categorized as heavy increased from 24% to 31% and for women it increased from 13% to 20%. The very heavy category increased from 4% to 10% for men and from 2% to 8% for women. The demographic/behavioural characteristics of participants are presented in Table 1, wherein the proportions of each characteristic have been determined both pre-change and post-change. There were notable changes in the very heavy group. After the recalculations, the very heavy group increased in the proportion of women (from 16% to 26%), those from the high SEP group (from 43% to 56%), those with a good diet (from 75% to 83%) and those with adequate physical activity levels (from 80% to 86%).

**Table 1.** Characteristics of Whitehall II participants by drinking category

	None	Former drinker	Moderate	Heavy	Very Heavy
Pre-change, *N* (Col %)	517	619	4198	1444	232
Years of age, mean (SD)	57 (6)	57 (6)	56 (6)	55 (6)	55 (6)
Sex					
Male	245 (47)	323 (52)	3021 (72)	1169 (81)	195 (84)
Female	272 (53)	296 (48)	1177 (28)	275 (19)	37 (16)
Ethnicity					
White	366 (71)	533 (86)	3936 (94)	1405 (97)	226 (97)
Non-white	151 (29)	86 (14)	262 (6)	39 (3)	6 (3)
Smoking					
Current	63 (12)	84 (14)	358 (9)	179 (12)	60 (26)
Former	107 (21)	215 (35)	1663 (40)	741 (51)	103 (44)
None	347 (67)	320 (52)	2177 (52)	524 (36)	69 (30)
SEP					
High	89 (17)	132 (21)	1853 (44)	820 (57)	100 (43)
Medium	242 (47)	307 (50)	1852 (44)	550 (38)	117 (50)
Low	186 (36)	180 (29)	493 (12)	74 (5)	15 (6)
Diet					
Good	399 (77)	506 (82)	3562 (85)	1204 (83)	173 (75)
Poor	118 (23)	131 (18)	636 (15)	240 (17)	59 (25)
Physical activity					
Active	389 (75)	483 (78)	3739 (89)	1312 (91)	186 (80)
Inactive	128 (25)	1360 (22)	459 (11)	132 (9)	46 (20)
Post-change, *N* (Col %)	As above	As above	3279	1943	652
Years of age, mean (SD)			56 (6)	55 (6)	55 (6)
Sex					
Male			2369 (72)	1531 (79)	485 (74)
Female			910 (28)	412 (21)	167 (26)
Ethnicity					
White			3038 (93)	1890 (97)	639 (98)
Non-white			241 (7)	53 (3)	13 (2)
Smoking					
Current			1747 (53)	803 (41)	220 (34)
Former			1244 (38)	943 (49)	320 (49)
None			288 (9)	197 (10)	112 (17)
SEP					
High			1321 (40)	1089 (56)	363 (56)
Medium			1519 (46)	746 (38)	254 (39)
Low			439 (13)	108 (6)	35 (5)
Diet					
Good			2745 (84)	1654 (85)	540 (83)
Poor			534 (16)	289 (15)	112 (17)
Physical activity					
Active			2918 (89)	1757 (90)	562 (86)
Inactive			361 (11)	186 (10)	90 (14)

Pre- and Post-change in wine conversion (Moderate = 1–168 g men and 1–112 g women, heavy = 169–407 g men and 113–287 g women, very heavy = 408+ men and 288+ women).

**Fig. 2.**
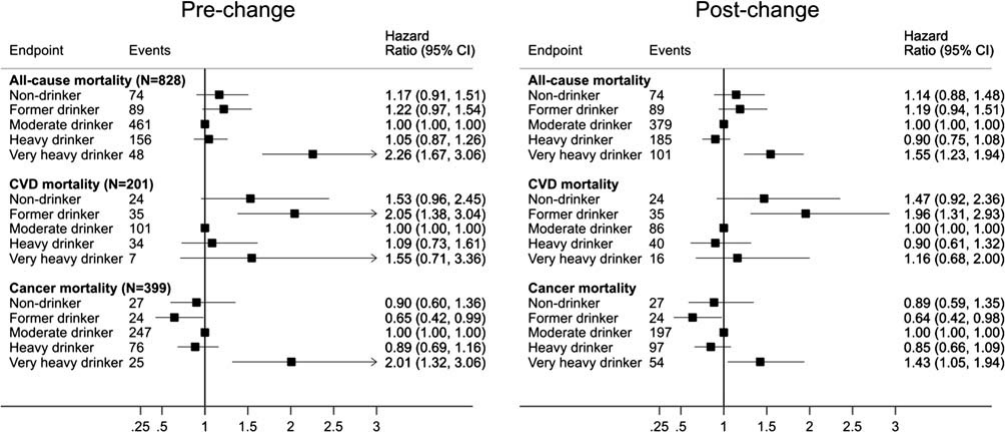
HRs (95% CIs) for all-cause, CVD and cancer mortality by drinking category pre- and post-change in wine conversion.

The HRs for mortality (from all causes, CVD and cancer) are presented in Fig. 2. During 115,844 person-years, 828 deaths occurred (201 ascribed to CVD and 399 to cancer). When the lower wine conversion was used, the HR for very heavy drinking compared to moderate drinking was 2.26 (95% CI 1.67–3.06) for all-cause mortality. This was reduced to 1.55 (95% CI 1.23–1.94) when the higher wine conversion was used (this equates approximately to a 46% reduction in estimated risk). The HR for very heavy versus moderate drinkers for CVD mortality reduced from 1.55 (95% CI 0.71–3.36) to 1.16 (95% CI 0.68–2.00) and for cancer mortality from 2.01 (95% CI 1.32–3.06) to 1.43 (95% CI 1.05–1.94). The HRs for heavy drinkers also reduced post-change, but were essentially non-significantly different to moderate drinkers before and after the higher alcohol content was implemented. The *I* ^2^ statistic ranged from 0% to 15.4% (indicating little or no heterogeneity for all comparisons—data not shown).

## Discussion

When applying a higher alcohol content conversion for wine consumption in the Whitehall II cohort study, the proportion of heavy/very heavy drinkers increased from 28% to 41% for men and 15% to 28% for women. ‘The significant increased risk of very heavy drinking (compared to moderate drinking) was present for death from all causes and cancer mortality before and after the change in wine conversion. However, the HRs were reduced when a higher wine conversion was used’. This may in part be explained by the more favourable profiles of those who consumed wine as shown by the increases in proportion of very heavy drinkers who were of high SEP, followed a good diet and maintained adequate physical activity levels. Furthermore, it is likely that this reduction in effect size is also partially attributable to the increase in the number of participants belonging to the very heavy drinking group after conversion (2.5- and 4-fold increases for men and women, respectively), improving the precision of the effect estimate as evidenced by narrower confidence intervals.

Since 1990, in the UK there was a shift from traditional European wines to those with higher alcohol content imported from South America and Australasia (‘New World’ wines) (Stead *et al.*, 2013). At the same time, the size of glass in which wine was served in licensed premises changed from a norm of 125 ml in 1990 to commonplace measures of 175 ml and 250 ml by 2010. Surveys and epidemiological studies using data collected in the 1990 onwards should revisit the conversion assumptions for alcohol content in glasses of wine. Using a conversion ratio of one wine glass to 8 g alcohol (1 unit of alcohol) is likely to result in a miscalculation of the true alcohol content. The implications of underestimating the alcohol content of drinks will undoubtedly have a knock-on impact on population-level drinking statistics, such as the mean amount of alcohol consumed on a weekly basis and the proportion of people in the population classified as drinking within or above sensible drinking guidelines. Moreover, we show, using data collected in the late 1990s, that not accounting for these historical changes in serving size and ABV of wine in terms of estimating the association between consumption categories and mortality is likely to have led to an overestimation of the detrimental effects of very heavy drinking.

Our study has several strengths including a large sample size and lengthy follow-up for mortality (~18 years). Unlike previous studies concerning wine conversion factors, we looked at the consequences of underestimation on mortality risk, not just change in alcohol intake. However, there are also several shortcomings. It is known that heavy drinkers are under-represented in population-level surveys; the drinkers in our sample are typically low to moderate consumers. Inferences must therefore be limited regarding particularly high levels of consumption. We did not address underestimation in terms of alcohol content in beer in this analysis; however, wine was the most common beverage, particularly among women. Nor did we consider misreporting of number of drinks (either intentional or unintentional). This is likely to differ by age, gender, drinking level and beverage choice (Livingston and Callinan, 2015). Drinks purchased from the off-trade (i.e. consumption taking place away from licensed premises) are also at risk of under-reporting as it has been demonstrated that people often pour larger servings than on-trade standards (Boniface *et al.*, 2013).

## Conclusion

This study extends previous work that has estimated the potential impact on ‘true’ alcohol intake of using a higher wine conversion to reflect increasing glass sizes and increasing wine strength. We are the first to demonstrate that, in a population-based cohort study, this translates into an overestimation of the health risks associated with very heavy drinking compared to moderate consumption. We propose that investigator-led cohorts need to revisit conversion factors based on more accurate estimates of alcohol content in wine glasses. Prospectively, researchers need to collect more detailed information on alcohol including serving sizes, strength and whether purchased off-trade/on-trade.

## Author Contributions

S.B. and A.B. conceived the research question. S.B. carried out the analysis. A.B. completed the first draft of the manuscript; S.B. and D.O.N. contributed to revisions of the manuscript. All authors saw and agreed on the final submitted manuscript. None of the authors have any conflict of interest to declare.
